# C2238 ANP gene variant promotes increased platelet aggregation through the activation of Nox2 and the reduction of cAMP

**DOI:** 10.1038/s41598-017-03679-9

**Published:** 2017-06-19

**Authors:** Roberto Carnevale, Pasquale Pignatelli, Giacomo Frati, Cristina Nocella, Rosita Stanzione, Daniele Pastori, Simona Marchitti, Valentina Valenti, Maria Santulli, Emanuele Barbato, Teresa Strisciuglio, Leonardo Schirone, Carmine Vecchione, Francesco Violi, Massimo Volpe, Speranza Rubattu, Sebastiano Sciarretta

**Affiliations:** 1 Department of Medico-Surgical Sciences and Biotechnologies, Sapienza University of Rome, 04100 Latina (LT), Italy; 2 Department of Internal Medicine and Medical Specialties, Sapienza University of Rome, 00161 Rome (RM), Italy; 3 Department of AngioCardioNeurology, IRCCS Neuromed, 86077 Pozzilli (IS), Italy; 4 Department of Imaging, Bambino Gesù Children Hospital, IRCCS, Rome, Italy; 5 Department of Experimental Medicine, Sapienza University of Rome, Rome, Italy; 6 Cardiovascular Center Aalst, Onze-Lieve-Vrouw Hospital, Aalst, Belgium; 7 Division of Cardiology, Department of Advanced Biomedical Sciences, University of Naples Federico II, Naples, Italy; 8 University of Salerno, Medicine and Surgery, Baronissi (Salerno), Italy; 9 Department of Clinical and Molecular Medicine, Sapienza University of Rome, Rome, Italy

## Abstract

Subjects carrying the C2238 variant of the atrial natriuretic peptide (ANP) gene have a higher occurrence of stroke and acute coronary syndrome, suggesting an increased predisposition to acute thrombotic events in these subjects. We evaluated for the first time the direct effects of mutant ANP (C2238/αANP) on platelet activation *in vitro* and in human subjects. *In vitro*, platelets were incubated with no peptide, with T2238/αANP (WT) or with C2238/αANP at different concentrations. C2238/αANP (10^−10^ M) induced higher collagen-induced platelet aggregation with respect to both control without ANP and T2238/αANP. This effect was even stronger at a higher concentration (10^−6^ M). Mechanistically, C2238/αANP significantly lowered platelet cAMP levels, increased ROS production and activated Nox2, with respect to both control and T2238/αANP. Forskolin, a cAMP activator, and sNOX2-tat, a Nox2 inhibitor, significantly reduced the pro-aggregant effects of C2238/αANP. *In vivo*, we found that platelet aggregation resulted to be higher in patients with atrial fibrillation carrying the C2238 ANP gene variant with respect to non-carriers. In conclusions, C2238/αANP promotes platelet aggregation through the activation of Nox2 and the reduction of cAMP.

## Introduction

Atrial natriuretic peptide (ANP) is a cardiac hormone which belongs to the family of natriuretic peptides. It is mainly secreted by atria in response to hypervolemia, cardiac dysfunction or cardiac hypertrophy, thereby eliciting diuretic, natriuretic and vasodilatory effects^1^. ANP also plays an important role in pleiotropic cardiovascular functions. It limits the development of cardiac hypertrophy and fibrosis and promotes endothelial cell survival and regenerative capacities^1^. However, accumulating lines of evidence demonstrated that the C2238 molecular variant of ANP (C2238/αANP) exerts detrimental vascular effects, predisposing to the development of cardiovascular diseases^1^. C2238/αANP is secondary to the T > C substitution at position 2238 of the ANP gene. Several studies demonstrated that subjects carrying the C2238 ANP gene variant have a higher risk of suffering a cardiovascular event^2–8^. We previously found that these subjects have a higher occurrence of stroke and acute coronary syndrome^2, 5, 6^. A previous study from Burnett’s group also showed that in an adult cohort from Olmsted County, which was followed up for 9 years, the C2238 ANP gene variant was associated with an increased incidence of cerebrovascular accidents and a higher prevalence of myocardial infarction^4^. In addition, previous work from Arnett’s group revealed a pharmacogenetic association of this ANP gene variant with cardiovascular disease outcomes in hypertensive subjects from the ALLHAT study^7^. Of note, the clinical impact of this ANP gene variant appears to be highly relevant, since its prevalence in the general population ranges from 15 to 23%. A specific therapeutic strategy to reduce the incidence of adverse cardiovascular events in subjects carrying the C2238 ANP gene variant is not available yet.

Experimental studies from our group demonstrated that C2238/αANP reduces survival and functional capacities of endothelial cells and vascular smooth muscle cells *in vitro*, through a deregulated activation of Type-C natriuretic peptide receptor (NPR-C)^9, 10^. This data indicates that C2238/αANP may predispose to the development of cardiovascular diseases by directly damaging vascular cells. However, we previously found that in patients with stable coronary artery disease, the presence of C2238 ANP gene allele is associated with a higher incidence of acute coronary syndromes, without influencing the extent and severity of coronary atherosclerotic plaques^5^. This data suggests that C2238 ANP minor allele might either increase atherosclerotic plaque rupture or alternatively, predispose to thrombosis by directly increasing platelet aggregation. However, the effects of C2238/αANP on platelet activity were never investigated.

Thus, the aim of our study was to evaluate the effects of C2238/αANP on platelet activation and aggregation *in vitro* and in patients with atrial fibrillation, a condition associated with thromboembolic events.

## Results

**Figure 1:**
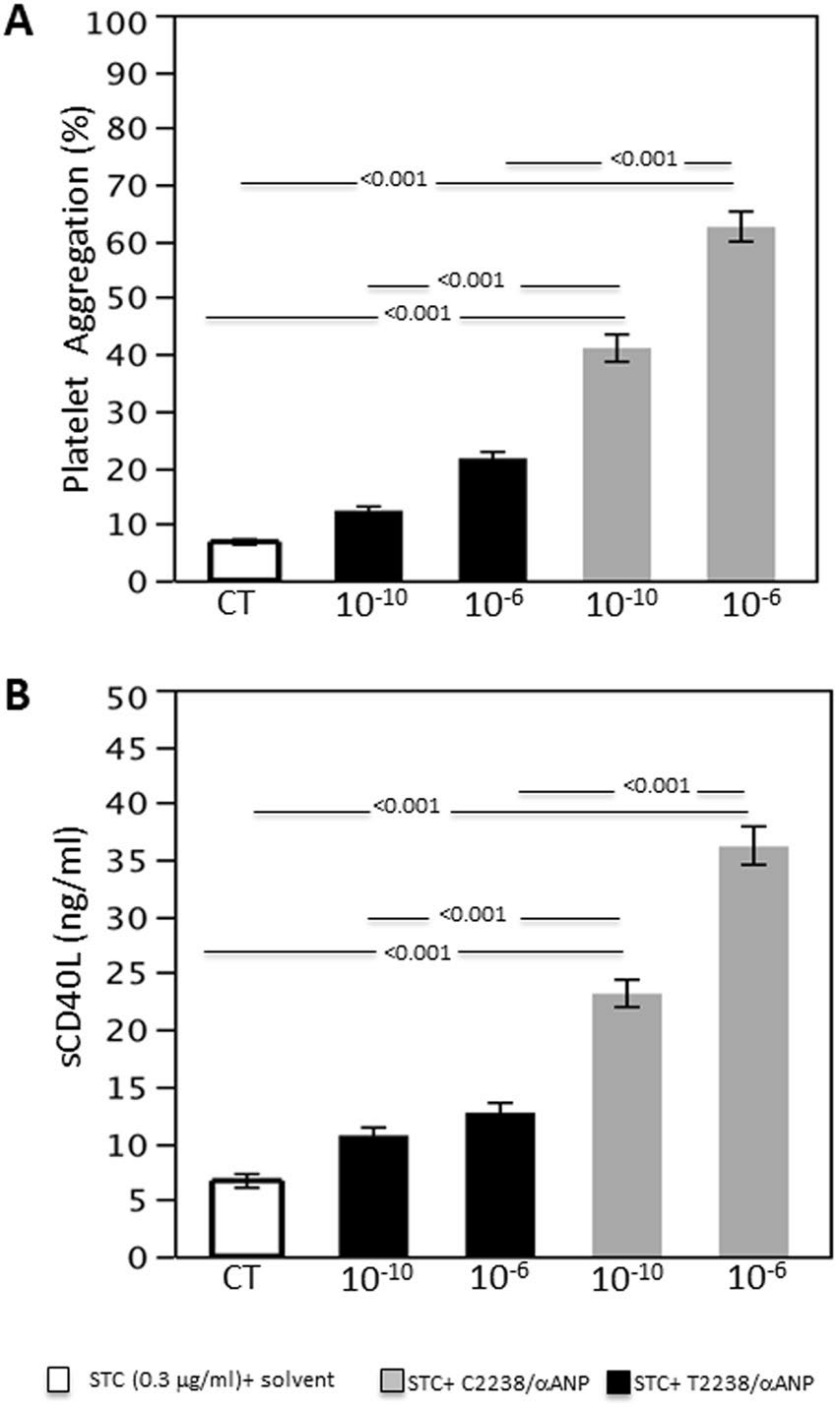
C2238/αANP induces platelet activation. (**A,B**) Platelets were incubated without the peptide or with either T2238/αANP or C2238/αANP at the specified concentration (10^−6^ M or 10^−10^ M) and the platelet activation was triggered by a sub-threshold concentration of collagen (0.3 µg/ml). Platelet aggregation (**A**) and the concentration of the soluble CD40 ligand released by platelets (**B**) were assessed. N = 12. The results were expressed as mean ± SEM. In all the groups, a similar volume of solvent was added.

In order to evaluate the effects of C2238/αANP on platelet activity, we incubated different concentrations of the peptide with human platelets from healthy subjects *in vitro* and then we measured platelet aggregation in response to a subthreshold concentration of collagen. We found that physiological (10^−10^ M) concentrations of C2238/αANP significantly induced platelet aggregation with respect to both control without ANP and T2238/αANP (Fig. 1A). This effect was even stronger at a higher ANP concentration (10^−6^ M), thus demonstrating that C2238/αANP induces platelet aggregation. Different concentrations of C2238/αANP also induced a higher release of soluble CD40 ligand, as compared to both control without ANP and T2238/αANP (Fig. 1B). This data further indicates that C2238/αANP significantly induces platelet activation. We found that T2238/αANP also modestly stimulates platelet aggregation, particularly at high concentration.

**Figure 2:**
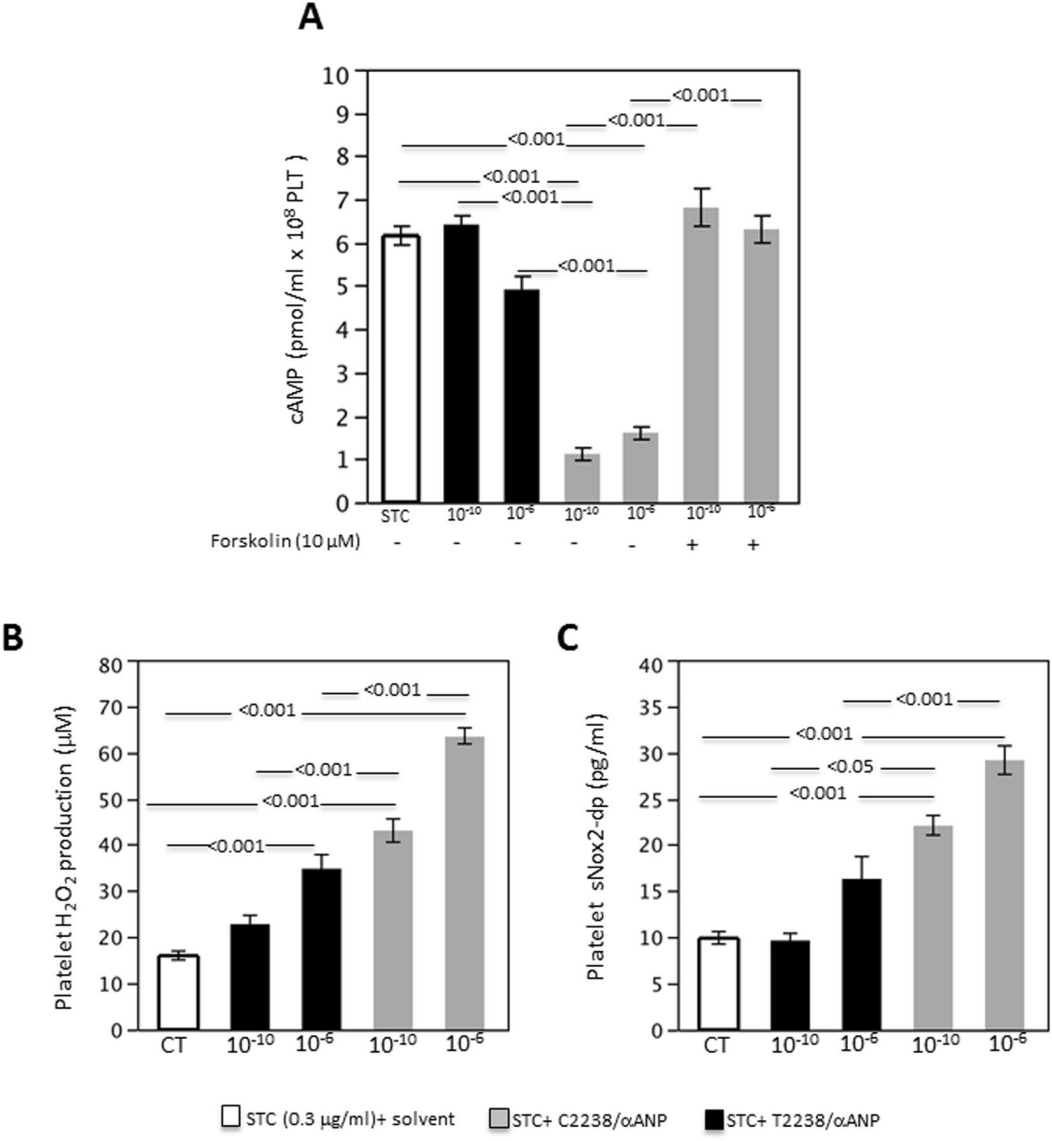
C2238/αANP reduces cAMP levels and promotes platelet oxidative stress. (**A–C**) Platelets were incubated without the peptide or with either T2238/αANP or C2238/αANP at the specified concentration (10^−6^ M or 10^−10^ M). Forskolin (10 µM) was also used where specified. After incubation and platelet stimulation with a sub-threshold concentration of collagen (0.3 µg/ml), intracellular cAMP levels (**A**), platelet hydrogen peroxide production (**B**) and platelet Nox2 activation (**C**) were assessed. N = 12. The results were expressed as mean ± SEM. In all the groups, a similar volume of solvent was added.

Then, we evaluated the mechanisms through which C2238/αANP induces platelet activation and aggregation. We previously demonstrated that C2238/αANP binds to NPR-C with a high affinity and activates it in a deregulated manner. On the other hand, it modulates NPR-A similarly to T2238/αANP^9^. NPR-C is coupled to a Gαi protein, which inhibits adenylate cyclase and reduces intracellular cyclic AMP (cAMP) levels when NPR-C is activated^11^. Therefore, we tested the effects of different concentrations of C2238/αANP on intracellular levels of cAMP. We found that C2238/αANP significantly reduces intracellular levels of cAMP with respect to control without ANP and T2238/αANP (Fig. 2A). These effects were reversed by forskolin, an activator of adenylate cyclase.

Our previous work also showed that C2238/αANP induces ROS accumulation and Nox2 activation in endothelial cells through the reduction of cAMP^9^. Therefore, we tested the effects of C2238/αANP on hydrogen peroxide and Nox2 activation in platelets. We found that C2238/αANP increases hydrogen peroxide release from platelets and activates Nox2, as indicated by higher release of its cleavage product, a marker of activation (Fig. 2B,C). This data indicates that C2238/αANP induces oxidative stress and Nox2 activation in platelets.

In order to test the hypothesis that a deregulated activation of NPR-C by C2238/αANP mediates its pro-aggregant effects on platelets, we evaluated whether C-ANF_4–23_, a selective NPR-C agonist, mimics the effects of C2238/αANP on platelet aggregation. First of all, we found that NPR-C is well expressed in human platelets (Supplementary Fig. S1). Interestingly, we found that platelet expression of NPR-C is higher in patients with a history of cardiovascular diseases and atrial fibrillation with respect to healthy subjects, thus indicating that NPR-C expression in platelets is directly correlated with cardiovascular risk status. Remarkably, we found that both physiological (10^−10^ M) and high concentrations (10^−6^ M) of C-ANF_4–23_ significantly induce platelet aggregation and CD40L expression with respect to both control without ANP and T2238/αANP (Supplementary Fig. S2), thus demonstrating that a selective activation of NPR-C is sufficient to induce platelet aggregation and activation. C-ANF_4-23_ also increased hydrogen peroxide production and Nox2 activation, with respect to both control without ANP and T2238/αANP (Supplementary Fig. S2).

**Figure 3:**
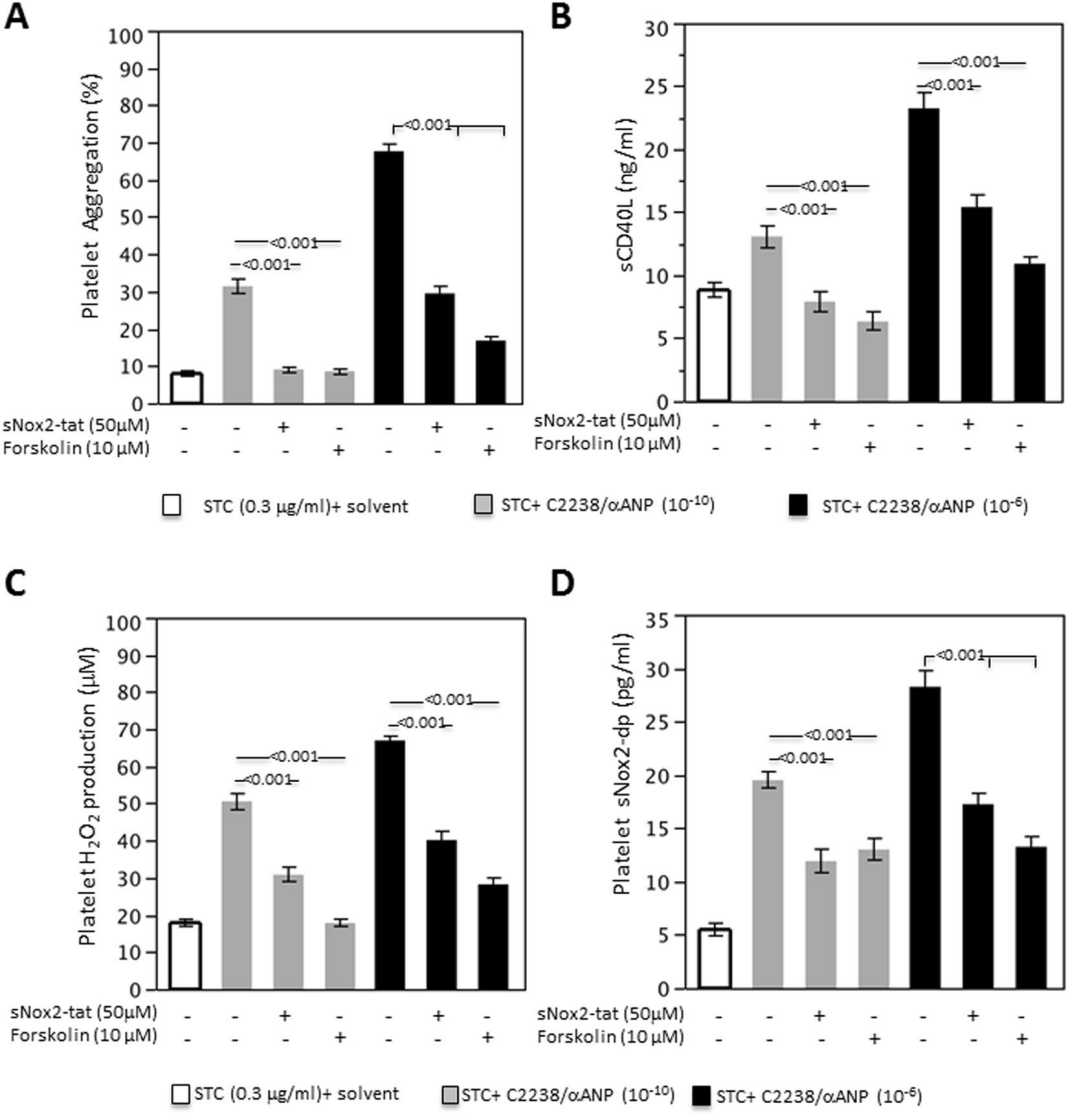
C2238/αANP induces platelet aggregation and oxidative stress by the inhibition of cAMP levels and activation of Nox2. (**A–D**) Platelets were incubated without the peptide or with C2238/αANP at the specified concentration (10^−6^ M or 10^−10^ M). Forskolin (10 µM) or sNOX2-tat (50 µM) were also used where specified. After incubation and platelet stimulation with a sub-threshold concentration of collagen (0.3 µg/ml), platelet aggregation (**A**), soluble CD40 ligand release (**B**), platelet hydrogen peroxide levels (**C**) and platelet Nox2 activity were assessed. N = 12. The results were expressed as mean ± SEM. In all the groups, a similar volume of solvent was added.

Then, we investigated whether lower intracellular levels of cAMP and higher ROS production and Nox2 activity are responsible for the increased platelet aggregation and activation induced by C2238/αANP. In fact, it is known that low intracellular levels of cAMP strongly induce platelet activation and aggregation^12^. Similarly, previous work demonstrated that Nox2 activation and high levels of ROS induce platelet aggregation^13–16^. We found that forskolin inhibited C2238/αANP-induced platelet aggregation and release of soluble CD40 ligand (Fig. 3A,B). In addition, forskolin inhibited C2238/αANP-induced hydrogen peroxide production and Nox2 activation in platelets (Fig. 3C,D). Similarly, Nox2 inhibition significantly blunted C2238/αANP-induced platelet activation and hydrogen peroxide production (Fig. 3A–C). sNox2-tat also efficiently reduced C2238/αANP-induced Nox2 activation (Fig. 3D). Overall, this data indicated that C2238/αANP induces platelet activation and aggregation through the reduction of intracellular cAMP levels and activation of Nox2. On the other hand, the effects of forskolin and sNOX2-tat on platelet aggregation induced by control and T2238/αANP were modest, consistently with the fact that cAMP and Nox2 are minimally modulated by these stimuli (Supplemental Fig. S3).

**Figure 4:**
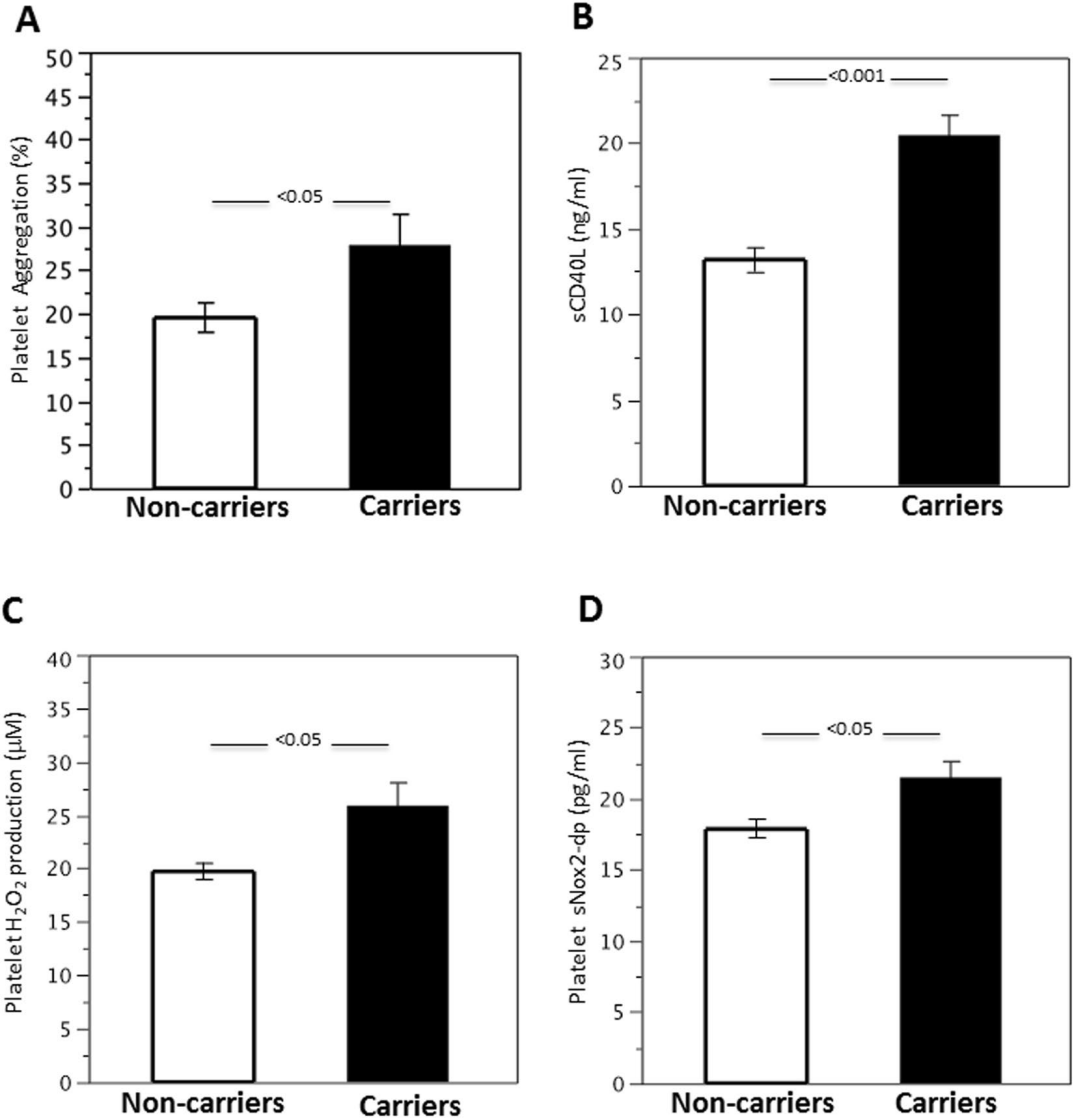
C2238 ANP gene variant is associated with increased platelet aggregation and oxidative stress. (**A–D**) Platelet aggregation (**A**), soluble CD40 ligand release (**B**), platelet hydrogen peroxide levels (**C**) and platelet Nox2 activity were assessed in subjects not carrying the variant and in subjects carrying the C2238 ANP gene variant. The results were expressed as mean ± SEM.

Finally, we wanted to extend this *in vitro* data to the clinical setting and tested whether subjects carrying the C2238 ANP gene variant display increased platelet aggregation. We enrolled a cohort of 195 patients with atrial fibrillation, a condition associated with high thromboembolic risk. Among these patients, 44 (39 heterozygous, 5 homozygous) were carriers of the C2238 minor allele. Main clinical characteristics did not differ significantly between carriers and non-carriers (Table 1). Serum levels of NT-proANP were lower in carrier subjects with respect to non-carriers, with a difference that was marginally significant (Table 1). On the other hand, no differences were observed regarding C-reactive protein levels (Table 1). Platelet count appeared to be similar in non-carrier and carrier subjects (non-carriers, 217.3 ± 58.6 SD × 10^9^/L vs. carriers, 204.4 ± 68.9 SD × 10^9^/L; p = 0.316). Nonetheless, we found that carriers of the C2238 variant displayed higher platelet aggregation with respect to non-carriers (Fig. 4A). They also showed higher release of CD40 ligand (Fig. 4B). Interestingly, we found that subjects carrying the C2238 variant also demonstrated higher hydrogen peroxide release and Nox2 activation in platelets (Fig. 4C,D), confirming that C2238/αANP induces ROS production and Nox2 activation in platelets *in vivo*. Of note, the highest rate of platelet aggregation and activation was observed in homozygous subjects, confirming the existence of a dose-dependent effect of C2238/αANP on platelet function (Supplementary Fig. S4).

**Table 1:** Baseline characteristics of the patients.

Variables	All (n = 195)	Non-carriers (n = 151)	Carriers (n = 44)	p
Age (years)	72.9 ± 9.6	73.4 ± 9.1	71.0 ± 10.9	0.143
Women (%)	38.5	35.8	47.7	0.163
CHA_2_DS_2_ VASc score	2.4 ± 1.5	2.5 ± 1.5	2.3 ± 1.5	0.444
Paroxysmal AF (%)	32.3	31.1	36.4	0.583
Time in therapeutic range (%)	61.4 ± 19.6	61.7 ± 19.0	60.3 ± 21.3	0.669
Arterial Hypertension (%)	86.2	85.4	88.6	0.804
Diabetes mellitus (%)	17.9	16.6	22.7	0.374
Heart failure (%)	14.4	12.6	20.5	0.222
History of ischemic stroke/TIA (%)	17.7	18.8	14.0	0.650
History of cardiac events (%)	19.3	19.5	18.6	0.900
Ejection fraction (%)	54.6 ± 8.4	54.9 ± 7.9	53.4 ± 10.3	0.378
Left atrium diameter (mm)	44.3 ± 6.0	44.3 ± 5.9	44.1 ± 6.2	0.889
C-reactive protein (mg/dl)	0.22 [0.13–0.41]	0.22 [0.12–0.40]	0.23 [0.15–0.50]	0.567
NT-proANP (pg/ml)	57.5 [50.7–85.4]	61.7 [51.0–89.3]	52.3 [48.1–70.0]	0.040
Antiplatelet therapy (%)	9.2	10.6	4.5	0.373
Diuretics (%)	41.0	41.1	40.9	0.986
Statins (%)	31.3	31.1	31.8	0.931
RAS inhibitors (%)	60.5	58.9	65.9	0.484

Continuous data are expressed as mean ± SD or as median with interquartile ranges (CRP and NT-proANP). RAS: Renin-Angiotensin System; TIA: transient ischemic attack; AF: atrial fibrillation.

## Discussion

In the present study, we explored the effects of a common ANP molecular variant on platelet aggregation and activation both *in vitro* and in a human cohort of patients with atrial fibrillation. We found that C2238/ αANP favors platelet aggregation and promotes ROS production in platelets through the reduction of intracellular cAMP levels and the activation of Nox2 *in vitro*. Importantly, we found that patients with atrial fibrillation carrying the C2238 ANP gene variant had increased platelet aggregation, activation and ROS production when compared to non-carriers.

The C2238 ANP gene variant consists of a mutation within the stop codon in exon 3, which leads to the addition of two arginine residues to the C-terminal portion of the mature form of ANP^1^. In recent years, the C2238 variant has emerged as a significant cardiovascular risk factor. In fact, subjects carrying the C2238 minor allele are at an increased risk for having a cerebrovascular accident or developing coronary artery disease^2–8^. In addition, human carriers also display early signs of endothelial dysfunction^9^. C2238/αANP directly induces vascular damage. In fact, we found that C2238/αANP induces apoptosis and oxidative stress in endothelial cells *in vitro* and reduces their angiogenetic properties. It also directly promotes endothelial dysfunction *ex vivo* ^9^. In addition, C2238/αANP induces vascular smooth muscle cell death, migration and increased contraction, which represent typical phenotypic changes occurring during the process of vascular remodeling^10, 17^. Mechanistically, all of these changes occur secondary to deregulated activation of NPR-C. In fact, NPR-C inhibition consistently attenuated the detrimental effects of C2238/αANP.

In the current work, we significantly extended these previous observations by demonstrating that C2238/αANP is also able to elicit direct pro-thrombotic effects on platelets by markedly increasing their aggregation and release of CD40 ligand. C2238/αANP also induced platelet oxidative stress. Of note, we also observed a proaggregant effect of wild type ANP at high concentrations, in accordance with a previous study by Loeb and Gear^18^ demonstrating that high levels of ANP are significantly associated with increased cardiovascular events^19, 20^. However, other investigations showed that wild type ANP may not affect platelet aggregation or it may even reduce it in some cases^21, 22^. The apparent divergence may be easily explained by differences in the experimental procedures among the studies, particularly regarding the platelet incubation time with the peptide and the method used to trigger platelet aggregation.

We found that C2238/αANP induces platelet aggregation by reducing cAMP levels. In fact, the restoration of cAMP levels by forskolin inhibited the pro-aggregant effects of the mutant peptide. Intracellular cAMP is universally recognized as a critical regulator of platelet aggregation^12^. High cAMP levels strongly inhibit platelet aggregation whereas reduced cAMP levels increase it. Of note, a reduction of platelet intracellular cAMP levels represents the mechanism mediating the pro-aggregant effects of several platelet agonists, particularly ADP^23, 24^. The inhibitory effects of cAMP appear to be mediated by PKA, which inhibits platelet activity at multiple levels keeping platelets in a resting state.

We believe that C2238/αANP inhibits cAMP levels by strongly activating NPR-C. NPR-C appears to be the most abundant natriuretic peptide receptor in platelets^25, 26^. NPR-C is coupled to a Gαi protein, which inhibits adenylate cyclase and cAMP production when the receptor is activated, thereby lowering intracellular cAMP levels. We previously observed that C2238/αANP binds to NPR-C with a higher affinity than wild type ANP and it activates this receptor in an exaggerated manner^9^. NPR-C knockdown rescues cAMP levels in endothelial cells incubated with C2238/αANP^9^. Therefore, it is very likely that C2238/αANP lowers platelet cAMP levels through the activation of NPR-C. Unfortunately, an inhibitor of NPR-C is not commercially available and it is not possible to knockdown NPR-C in platelets and then test aggregation. Therefore, the only feasible way to dissect the specific role of NPR-C in the regulation of platelet reactivity was to test the effects of its agonist. We found that C-ANF_4–23_, a selective NPR-C agonist, mimics the effects of C2238/αANP on platelet aggregation. This data is consistent with the evidence that ADP, a strong platelet agonist, reduces intracellular cAMP levels and induces platelet aggregation by activating the P2Y2 receptor^23, 24^, which is also coupled to a Gαi protein.

Of note, we found that platelet NPR-C expression level is higher in subjects with a history of cardiovascular diseases and atrial fibrillation with respect to healthy subjects, thereby suggesting that subjects at high cardiovascular risk are in general more vulnerable to the effects of C2238/αANP. Future studies are warranted to investigate whether an increased platelet expression of NPR-C in subjects at high cardiovascular risk contribute to the higher platelet reactivity and higher incidence of acute cardiovascular events observed in these subjects.

The proaggregant effects of C2238/αANP appears to also be mediated by Nox2 activation^13–16^. Nox2 is a major NADPH oxidase isoform in the cardiovascular system^27^. Nox2 is activated in response to stress thereby promoting ROS production and cardiovascular damage^27^. Our previous work demonstrated that Nox2 is also abundant in platelets, in which it regulates aggregation^13–16^. Nox2 was shown to be activated in platelets by cardiovascular risk factors, such as diabetes and dyslipidemia, thereby promoting superoxide generation and platelet aggregation. On the other hand, Nox2 inhibition was shown to reduce platelet aggregation^14^. ROS appear to activate platelets through calcium mobilization, reduction of nitric oxide levels and isoprostane production. In our study, we found that C2238/αANP promotes platelet activation of Nox2 *in vitro*. In addition, platelets from subjects carrying the C2238 ANP gene variant also displayed an increased Nox2 activation and ROS levels. Importantly, we found that pharmacological Nox2 inhibition significantly reduces platelet aggregation induced by C2238/αANP. This result is consistent with our previous data in endothelial cells, in which we observed that C2238/αANP induces ROS production and Nox2 activation^9^ and that NADPH oxidase inhibition partially rescues the detrimental effects of the mutant peptide^9^. Of note, forskolin inhibited C2238/αANP-induced ROS production and Nox2 activation in platelets. This evidence indicates that reduced levels of cAMP in response to C2238/αANP exposure contribute to Nox2 activation. This data is also consistent with previous results demonstrating that reduced cAMP levels promote NADPH oxidase activation^9, 11, 28, 29^.

We believe that our work has several clinical implications. Currently, there is no specific treatment able to reduce the increased cardiovascular risk of subjects carrying the C2238 ANP gene variant. Nox2 and ROS production might represent a potential therapeutic target. Nox2 inhibition would also reduce vascular oxidative stress and dysfunction induced by C2238/αANP. It should be pointed out that Nox2 inhibition should be partial and not complete, so that the systemic physiological functions exerted by Nox2 can still be preserved.

In addition, it is known that subjects with atrial fibrillation are prone to suffer from thromboembolism and cardiovascular events^30^. Previous work demonstrated that atrial fibrillation is associated with enhanced platelet activity, conferring a high risk of major adverse cardiovascular events^31^. In addition, Nox2-dependent ROS production was found to be associated with atherosclerotic ischemic complications in the setting of atrial fibrillation^32^. We think that medical attention should be devoted to subjects affected by atrial fibrillation carrying the C2238 gene variant since they may have an increased risk to incur thromboembolic events. It is possible that carriers affected by atrial fibrillation may benefit also from a therapy with platelet aggregation inhibitors, although this hypothesis needs to be tested in future studies.

Finally, future studies are needed to study the impact of C2238 variant on platelet aggregation in subjects with coronary artery disease, particularly those under treatment with antiplatelet drugs. In fact, the level of platelet inhibition reached by standard antiplatelet drug therapy may not be sufficient in subjects carrying the C2238 minor allele.

Our study demonstrated that C2238/αANP is associated with an increased platelet activation through the stimulation of Nox2 and the reduction of cAMP. These data suggest that C2238/αANP may induce the development of cardiovascular diseases not only inducing vascular damage and favoring atherosclerotic plaque rupture, but also by directly increasing platelet aggregation and inducing atherothrombotic events. We believe that Nox2 inhibition might represent a potential therapeutic intervention to reduce the cardiovascular risk in subjects carrying the C2238 ANP gene variant.

## Methods

### Platelet Aggregation Studies

In order to obtain platelet-rich plasma (PRP), citrated blood samples were centrifuged for 15 minutes at 180 g. To avoid leukocyte contamination, only the top 75% of the PRP was collected according to Pignatelli *et al*.^14^.

For the *in vitro* study, PRP samples from healthy subjects were pre-incubated (30 minutes at 37 °C) with different ANP peptides and inhibitors. In order to investigate the mechanisms of C2238/αANP-induced platelet activation, we used forskolin (10 µM), which actives adenylate cyclase, sNOX2-tat (AnaSpec, 50 µM), which inhibits NADPH oxidase activation and C-ANF_4–23_, a selective agonist of NPR-C (10^−10^ and 10^−6^ M). Synthetic wild type αANP (T2238/αANP) and mutant αANP (C2238/αANP) were purchased from Primm (codes 201409-00026 and 201409-00027 for C2238/αANP and T2238/αANP, respectively) and used at different concentrations which were achieved by diluting the peptide in water (10^−10^ and 10^−6^ M). In the control groups not treated with ANP peptides, the same volume of water was always added, as a control. Platelet number was the same for all the experimental groups and aggregation was induced by a sub-threshold concentration (STC) of collagen (Mascia Brunelli, 0.3 µg/mL in water) for 10 minutes. Platelet aggregation was measured as previously described^33^ with a Light Transmission Aggregometry (Cronolog) and the STC concentration of agonists was defined as the highest concentration that elicited <15% platelet aggregation. After activation, samples were centrifuged for 3 minutes at 3000 g.

Citrated blood and PRP samples were obtained from healthy subjects without cardiovascular diseases, who were enrolled in Policlinico Umberto I – Sapienza University of Rome, Italy, according to guidelines and regulations. The study protocol was approved by the local ethical board of Sapienza University of Rome and was conducted according to guidelines and regulations. Informed consent was obtained from all subjects.

For the *in vivo* study, platelets were obtained from subjects with atrial fribrillation, as described in the below *Study population* section. After isolation, platelet aggregation was induced by collagen (Mascia Brunelli, 2 µg/mL).

Supernatants were stored at −80 °C for analysis of sNox2-dp, sCD40L, H_2_O_2_ and cAMP. Intra-assay and inter-assay coefficients of variation were 5.7% and 6.8%, respectively.

### Measurement of C-reactive protein

The measurement of high-sensitivity C-reactive protein (CRP) was performed by the laboratory of D.E.A of Policlinico Umberto I – Sapienza University of Rome by using a nephelometric assay. The values were expressed as mg/dL.

### Measurement of NT-proANP

Plasma NT-proANP levels were assessed with a commercially-available ELISA kit (Elabsciences) and expressed as pg/ml. Intra-assay and inter-assay coefficients of variation were < 10%.

### Measurement of platelet sCD40L

Platelet levels of sCD40L were measured with the use of a commercial immunoassay (DRG International) and expressed as ng/ml. Intraassay and interassay coefficients of variation were 5% and 7% respectively.

### Measurement of H_2_O_2_ production

Hydrogen peroxide (H_2_O_2_) was evaluated by a Colorimetric Detection Kit (Arbor Assays) and expressed as μmol/L. Intra-assay and inter-assay coefficients of variation were 2.1% and 3.7% respectively.

### Assay of platelet Soluble Nox2-derived Peptide

Extracellular levels of soluble Nox2-derived peptide (sNox2-dp), a marker of NADPH oxidase activation, were detected by ELISA as previously described^15^. The peptide was recognized by the specific monoclonal antibody against the amino acidic sequence (224–268) of the extra membrane portion of Nox2 (catalytic core of NADPH oxidase), which is released in the medium upon platelet activation.

Values were expressed as pg/ml; intra-assay and inter-assay coefficients of variation were 5.2% and 6%, respectively.

### Measurement of cAMP in platelets exposed to wild-type or mutant ANP

cAMP measurement was performed with the specific enzymeimmunoassay Biotrak (EIA) System (Amersham), following the manufacturer’s instructions.

### Study population

We included 195 patients with non-valvular atrial fibrillation, who were treated with oral vitamin K antagonists (INR target 2.5), based on the CHA_2_DS_2_-VASc score^34^. The level of anticoagulation in these subjects was evaluated by the time in therapeutic range (TTR)^35^. Exclusion criteria were patients with prosthetic heart valves, severe valvulopathies, severe cognitive impairment, chronic infections, autoimmune systemic disease, active cancer and liver insufficiency (e.g. cirrhosis). At baseline, each patient provided written informed consent and patient’s anthropometric data and medical history were recorded. Baseline NT-proANP, platelet count and high-sensitivity C reactive protein were also measured. Cardiovascular risk factors, such as arterial hypertension, diabetes mellitus^36^ and heart failure^37^ were defined according to the currently used international definitions. The study protocol was approved by the local ethical board of Sapienza University of Rome and was conducted according to guidelines and regulations. Informed consent was obtained from all subjects.

### Genetic analysis

For the analysis of C2238 ANP allele carrier status, genomic DNA was extracted from peripheral whole blood by a commercially available kit (QIAamp DNA Blood Mini Kit, Qiagen) and stored at −20 °C until genotyping.

Genotyping was carried out by using 50 ng of genomic DNA in a total volume of 20 µl containing 1 µl of preformed Taqman assay 20X and 10 µl of Genotyping Master Mix 2X (Applied Biosystems, Foster City, CA). PCR reactions were carried out on a ViiA 7 PCR System (Applied Biosystems) at 95 °C for 10 min followed by 40 cycles of denaturation at 95 °C for 15 sec and annealing at 60 °C for 1 min. An electronic data file was generated, containing genotypes and the quality value.

### Immunoblot analysis

NPR-C expression levels were measured in platelets isolated from 5 healthy subjects (3 males, 2 females, age 51.2 ± 12.4) and 5 patients with atrial fibrillation and history of cardiovascular diseases (3 males, 2 females, age 57.8 ± 5.2). Platelet pellets were suspended in a 2X Lysis buffer (5 mM EDTA, 0.15 mol NaCl, 0.1 mol Tris pH 8.0, 1% triton and 10 μg/ml of protease and phosphatase inhibitors cocktail). Equal amounts of protein (30 μg/lane) estimated by Bradford protein assay were solubilized in a 2X Leammli sample buffer containing 20% of 2-mercaptoethanol. Proteins were separated by SDS-PAGE on a 10% polyacrylamide gel and then electro-transferred to nitrocellulose membranes. After protein transfer, the membranes were blocked in 1X TBST solution with 5% milk at room temperature for 1 h. After blocking, membranes were incubated with rabbit anti-NPR-C antibody (Santacruz) in 1X TBST with 5% BSA (1:500) overnight at 4 °C. Then, the membranes were incubated with a secondary antibody (Santa Cruz Biotechnology, 1:5000) and the immune complexes were detected by enhanced chemiluminescence substrate. Densitometric analysis of the bands was performed by using Image J software.

### Reagents

All materials were from Sigma Aldrich unless otherwise specified.

### Transthoracic echocardiography (TTE)

At baseline, each patient was examined by resting TTE; left atrium antero-posterior diameter (mm) and systolic left ventricular function as assessed by ejection fraction (EF %, Simpson method) were measured according to international guidelines^38, 39^.

### Statistical analysis

Kolmogorov-Smirnov test was used to assess distribution of continuous variables, when appropriate. Continuous variables are expressed as mean ± SEM unless otherwise specified. Student *t* test or Mann-Whitney test was used to compare means or medians of two independent groups, when appropriate.

When more than two groups were compared one-way ANOVA test was used and Bonferroni post-hoc test was performed for intergroup comparisons. Categorical variables were compared by using the chi-squared test.

Statistical significance was set at a p value lower than <0.05. Analyses were performed with SPSS statistical software (SPSS Inc, Chicago, IL, version 20).

## Electronic supplementary material

Supplementary Material
