# Supplementary material for: C2238 ANP gene variant promotes increased platelet aggregation through the activation of Nox2 and the reduction of cAMP
Source: Sci Rep. 2017 Jun 19;7:3797. doi: 10.1038/s41598-017-03679-9 (PMC5476672; doi:10.1038/s41598-017-03679-9)

SUPPLEMENTARY MATERIAL

C2238 ANP gene variant promotes increased platelet aggregation through the activation of Nox2 and the reduction of cAMP

Roberto Carnevale, PhD, Pasquale Pignatelli, MD, Giacomo Frati, MD, Cristina Nocella, MS, Rosita Stanzione, PhD, Daniele Pastori, MD, Simona Marchitti, BS, Valentina Valenti, MD, Maria Santulli, PhD, Emanuele Barbato, MD, Teresa Strisciuglio, MD, Leonardo Schirone, MS, Carmine Vecchione, MD, Francesco Violi, MD, Massimo Volpe, MD, Speranza Rubattu, MD, Sebastiano Sciarretta, MD, PhD

Supplementary Figure Legends

Supplementary Figure 1. NPR-C expression level in human platelets
Platelets were isolated from healthy subjects (N=5) or patients with atrial fibrillation and history of cardiovascular diseases (N=5). NPR-C expression was evaluated by immunoblot analysis. The results were expressed as mean ± SEM.

Supplementary Figure 2. NPR-C activation promotes platelet aggregation
A-D. Platelets from healthy subjects were incubated with C-ANF4-23 or T2238/αANP at the specified concentrations. Platelet aggregation (A), soluble CD40 ligand release (B), platelet hydrogen peroxide levels (C) and platelet Nox2 activity (D) were assessed. The results were expressed as mean ± SEM.

Supplementary Figure 3. C2238/αANP induces platelet aggregation through the reduction of intracellular cAMP levels and activation of Nox2
A-B. Platelet aggregation was performed in platelets from healthy subjects incubated with or without the specified ANP peptides, in the presence or not of sNox2-tat at the specified concentrations (A). Platelet aggregation was performed in platelets from healthy subjects incubated with or without the specified ANP peptides, in the presence or not of forskolin at the specified concentrations (B). N=5. The results were expressed as mean ± SEM.

Supplementary Figure 4. Effects of C2238 ANP gene variant on platelet aggregation and ROS levels
A-D. Platelet aggregation (A), soluble CD40 ligand release (B), platelet hydrogen peroxide levels (C) and platelet Nox2 activity (D) were assessed in subjects not carrying the variant (WT, N=151) and in subjects heterozygous (HET, N=39) and homozygous (HOM, N=5) with regard to the C2238 ANP gene variant. The results were expressed as mean ± SEM.

Supplementary Figure S1

A

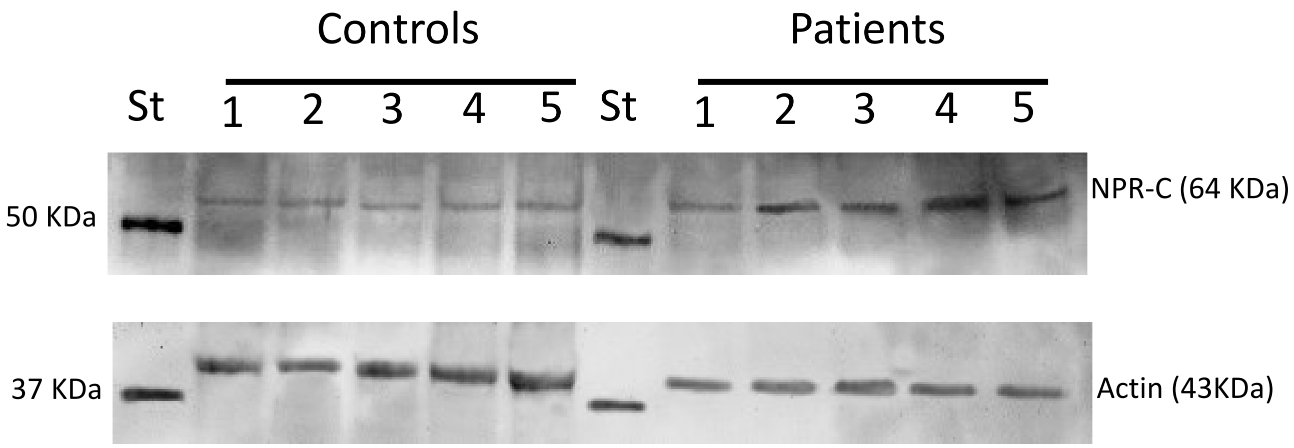

B

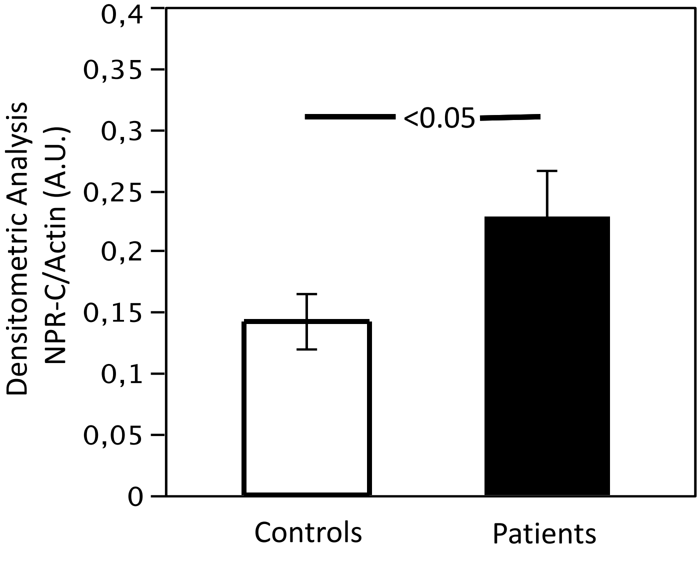

Supplementary Figure S2

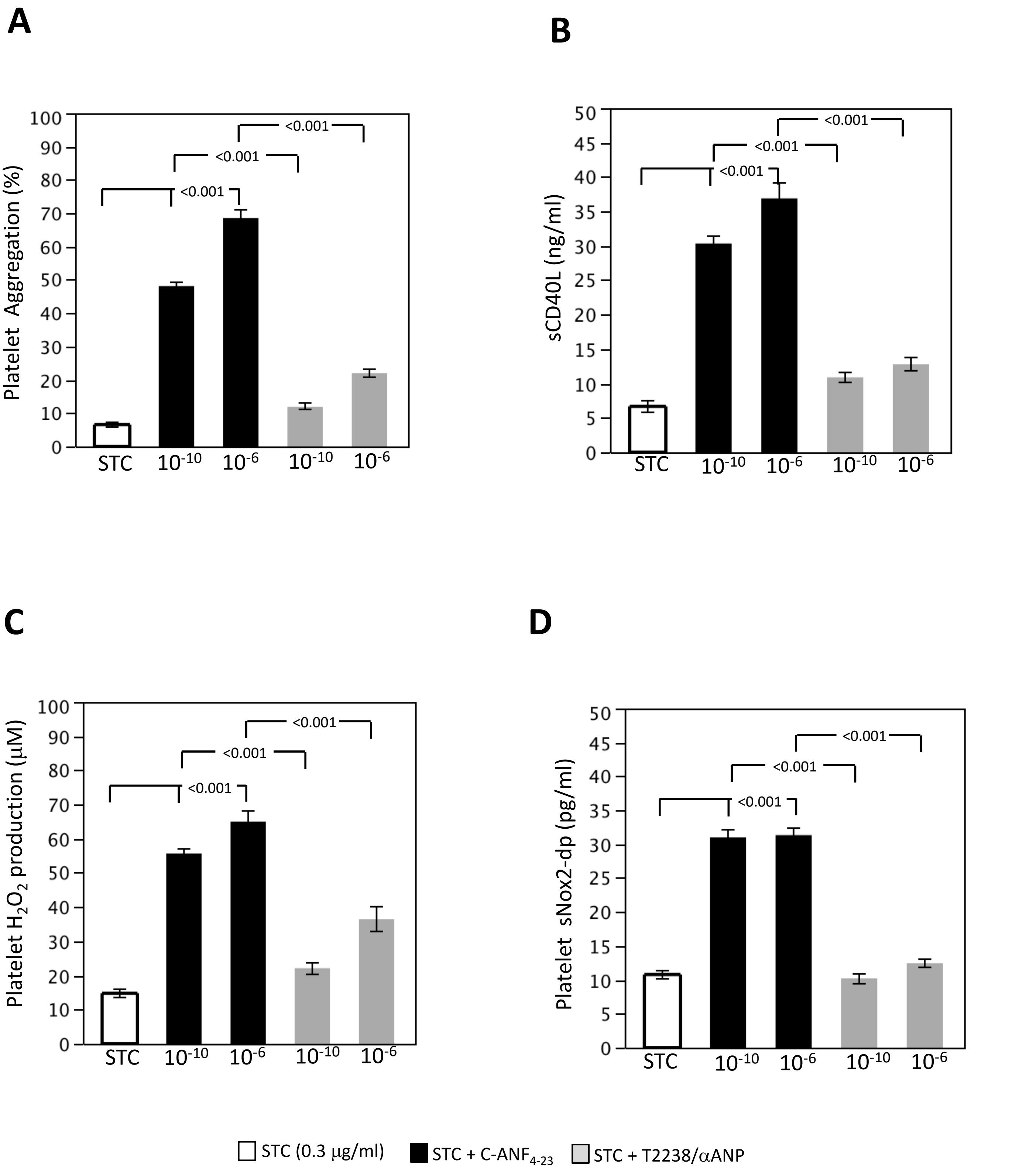

Supplementary Figure S3

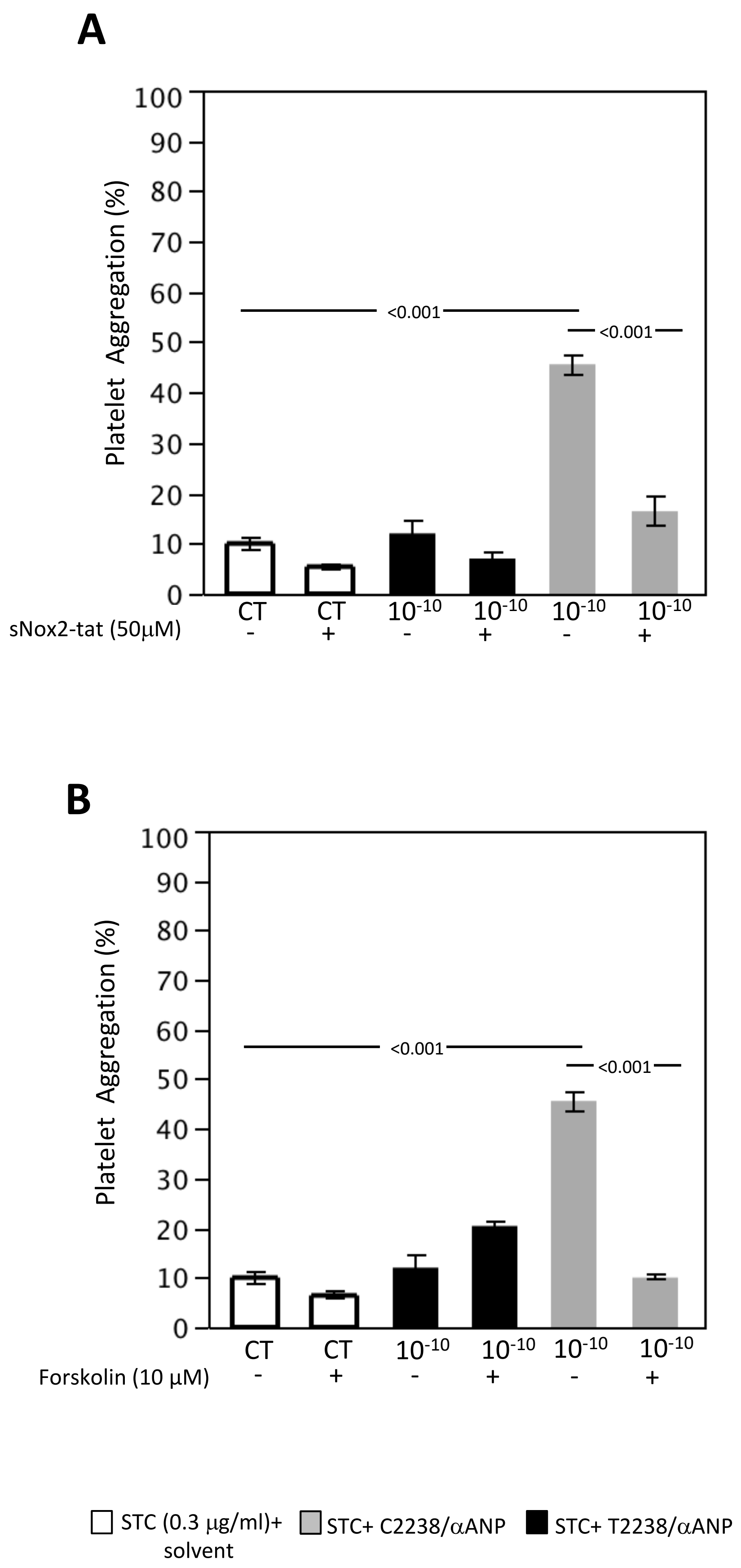

Supplementary Figure S4

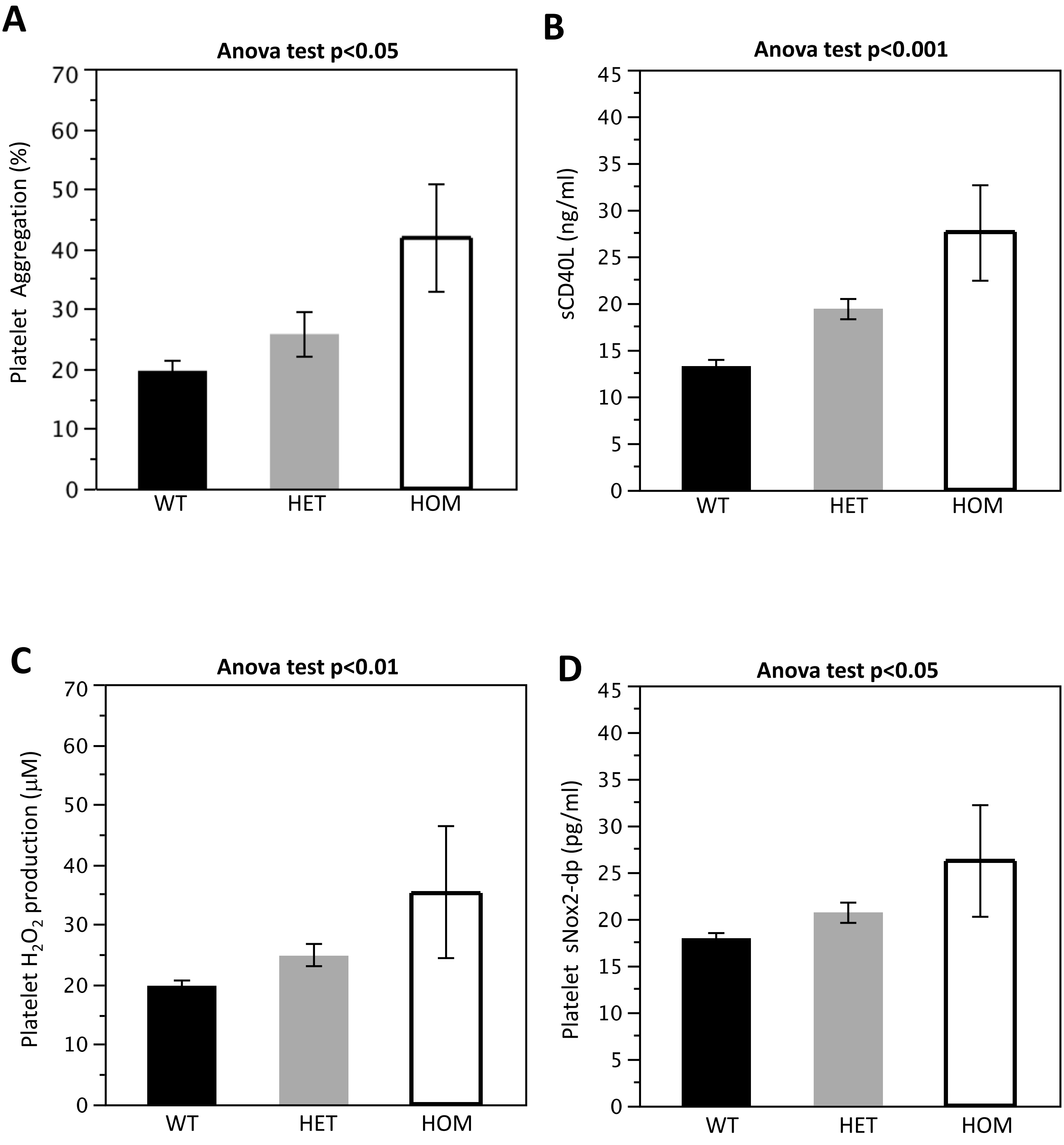

Supplement: Supplementary file 1 — Supplementary Material [file 41598_2017_3679_MOESM1_ESM.pdf]
